# Dual Quantification of Skeletal Muscle Perfusion and Metabolism in a Porcine Model of Peripheral Artery Disease Using Multiparametric ^18^F-FDG PET Imaging

**DOI:** 10.1007/s11307-026-02106-8

**Published:** 2026-05-01

**Authors:** Ting-Heng Chou, Mahboubeh Nabavinia, Eleanor T. Rimmerman, Corrin Mansfield, Kumudha Narayana Musini, Nguyen K. Tram, Mitchel R. Stacy

**Affiliations:** 1 Center for Regenerative Medicine, The Research Institute at Nationwide Children’s Hospital, 575 Children’s Crossroad, Columbus, OH WB413343215 USA; 2 Department of Exercise and Health Science, National Taipei University of Nursing and Health Sciences, Taipei City, Taiwan; 3 Interdisciplinary Biophysics Graduate Program, Ohio State University, Columbus, OH USA; 4 Division of Vascular Diseases and Surgery, Department of Surgery, Ohio State University College of Medicine, Columbus, OH USA

**Keywords:** Perfusion imaging, Peripheral artery disease, PET, Fluorodeoxyglucose, Skeletal muscle, Metabolism

## Abstract

**Background:**

A standard imaging strategy for quantifying skeletal muscle perfusion in peripheral artery disease (PAD) does not exist, and the widespread use of PET imaging for this purpose has traditionally been limited by the need for onsite production of short half-life perfusion radioisotopes. Therefore, this study investigated the feasibility of multiparametric PET imaging with commercially available fluorine-18 (^18^F)-fluorodeoxyglucose (FDG) for the quantification of skeletal muscle perfusion and metabolism in a porcine model of PAD.

**Methods:**

Eight Yorkshire pigs underwent 60-min dynamic ^18^F-FDG PET imaging under resting conditions immediately following unilateral surgical ligation of the femoral artery and 2 weeks after arterial occlusion. Calf muscle perfusion was computed using 1-compartment modeling of the first 2.5 min of PET data acquisition, and the metabolic rate of glucose (MRGlu) was computed using 3-compartment modeling of the entire 60-min dataset. Two weeks after arterial occlusion, the gastrocnemius muscle was harvested to compare microvascular density between ischemic and control hindlimbs.

**Results:**

Calf perfusion and MRGlu were significantly reduced following peripheral artery occlusion and recovered to control levels 2 weeks later. Recovery of perfusion and metabolism in calf skeletal muscle coincided with a significant increase in calf muscle capillary density 2 weeks after arterial occlusion.

**Conclusions:**

This study demonstrates the novel use of dynamic, multiparametric ^18^F-FDG PET/CT imaging for quantifying ischemia-induced alterations in skeletal muscle perfusion and metabolism, providing a unique comprehensive approach for evaluating PAD pathophysiology and creating opportunities for monitoring treatment responses to emerging therapeutics.

## Introduction

Peripheral artery disease (PAD) is a progressive atherosclerotic disease that affects the lower extremities of more than 12 million Americans and 230 million people worldwide [1], significantly impairs quality of life, and increases risk of lower extremity amputation and death [2]. Medical therapy for limb salvage in PAD is largely focused on restoring limb blood flow using endovascular and open surgical revascularization techniques; however, standard non-invasive clinical tools (e.g., ankle-brachial index, toe-brachial index) and imaging techniques (e.g., Duplex ultrasound, x-ray computed tomography (CT) and magnetic resonance angiography) for evaluating PAD patients have largely focused on measuring lower extremity hemodynamics or arterial anatomy and do not commonly quantify tissue-level functional parameters, such as deficits in skeletal muscle perfusion or metabolism, that could improve characterization of the pathophysiology associated with varying stages of PAD severity, allow for enhanced monitoring of disease progression, and regionally assess responses to conventional and emerging PAD therapies. Thus, there is a need for a functional imaging approach that quantifies lower extremity skeletal muscle pathophysiology in PAD beyond the scope of standard practices.

In recent years, nuclear imaging-based approaches have demonstrated utility for evaluating skeletal muscle perfusion in PAD. Specifically, hybrid single photon emission computed tomography (SPECT)/computed tomography (CT) imaging has demonstrated utility for non-invasively detecting volumetric regional abnormalities in resting foot perfusion in PAD patients [3, 4], quantifying regional improvements in foot perfusion in response to endovascular revascularization [5], and identifying patients at high risk of amputation following peripheral interventions [6]. Positron emission tomography (PET) imaging has also shown utility for quantifying absolute measures of skeletal muscle perfusion in PAD patients [7–9]. However, to date, SPECT imaging in PAD has been limited to assessing only relative perfusion, and radioisotopes traditionally used for PET perfusion imaging (e.g., rubidium-82, oxygen-15 water) possess relatively short half-lives (< 5 min) that require on-site production and rapid administration to patients, which necessitates well-trained radiopharmacy staff, increases imaging costs, and limits the scalability of the approach to the broader vascular medicine and PAD communities. To address these limitations, our team recently pre-clinically validated and clinically translated a fast 2.5-min dynamic PET imaging approach that quantifies absolute measures of skeletal muscle perfusion in PAD using a commercially available ^18^F-labeled radionuclide (^18^F-sodium fluoride, NaF), which possesses a longer half-life (109.7 min) and allows for unit dose delivery from nearby vendors of radiopharmaceuticals [10]. Given that dynamic ^18^F-NaF PET imaging has demonstrated potential for quantifying resting measures of calf muscle perfusion in PAD, in the present study, we sought to further assess the utility of a more readily available and widely used ^18^F-labeled PET radionuclide, ^18^F-fluorodeoxyglcuose (FDG), for quantifying abnormalities in skeletal muscle perfusion in the setting of limb ischemia. Considering that ^18^F-FDG PET imaging has previously been used to quantify skeletal muscle metabolism in PAD patients [11], we also sought to investigate if multiparametric analysis of a dynamic ^18^F-FDG PET scan could provide quantification of both skeletal muscle perfusion and metabolism using single dose administration of ^18^F-FDG. We hypothesized that skeletal muscle perfusion could be quantified using the first 2.5 min of PET data, while muscle metabolism could also be measured in the same PET field-of-view using the entirety of a 60-min dynamic PET acquisition. Therefore, the present study pre-clinically tested the feasibility of multiparametric ^18^F-FDG PET imaging for comprehensive physiological evaluation of abnormalities in skeletal muscle perfusion and metabolism in the setting of PAD using a porcine model of hindlimb ischemia.

## Materials and Methods

The data that support the findings of this study are available from the corresponding author upon reasonable request.

### Large Animal Model of Hindlimb Ischemia

Male Yorkshire pigs (N = 8; 10.1 ± 0.4 kg) approximately 4 weeks of age underwent surgical cutdown and permanent ligation of the right femoral artery to induce unilateral hindlimb ischemia. A full description of surgical preparation and techniques for this animal model have been previously described in prior publications [10, 12]. The animal protocol was reviewed and approved by the Institutional Animal Care and Use Committee of Nationwide Children’s Hospital and was in compliance with the Association for Assessment and Accreditation of Laboratory Animal Care International policies. All procedures followed the National Institutes of Health Guide for the Care and Use of Laboratory Animals. The use of radioisotopes and all imaging procedures were approved by the Radiation Safety Committee of Nationwide Children’s Hospital. The ARRIVE guidelines and regulations were followed for reporting of animal use and study results [13].

### ^18^F-FDG PET/CT Imaging Protocol

Animals were fasted for a minimum of 8 h prior to PET/CT imaging. The jugular vein and carotid artery were percutaneously accessed, and 4-F polyethylene catheters (Cook Medical, Bloomington, IN) were placed to allow for venous administration of ^18^F-FDG for PET imaging and continuous arterial blood sampling during PET imaging. To maintain consistent anesthesia levels during PET/CT imaging, propofol was infused at 10–30 mg/kg/hour through an ear vein catheter. The depth of anesthesia was monitored every 15 min by evaluating blink reflex and jaw tone. Physiological parameters including blood pressure, heart rate, oxygen saturation, and body temperature were monitored continuously for the duration of PET/CT imaging sessions (IntelliVue MP30; Philips Healthcare, Andover, MA), with body temperature maintained above 37 degrees Celsius to control for the potential influence of temperature on measures of skeletal muscle perfusion and metabolism.

**Fig. 1:**
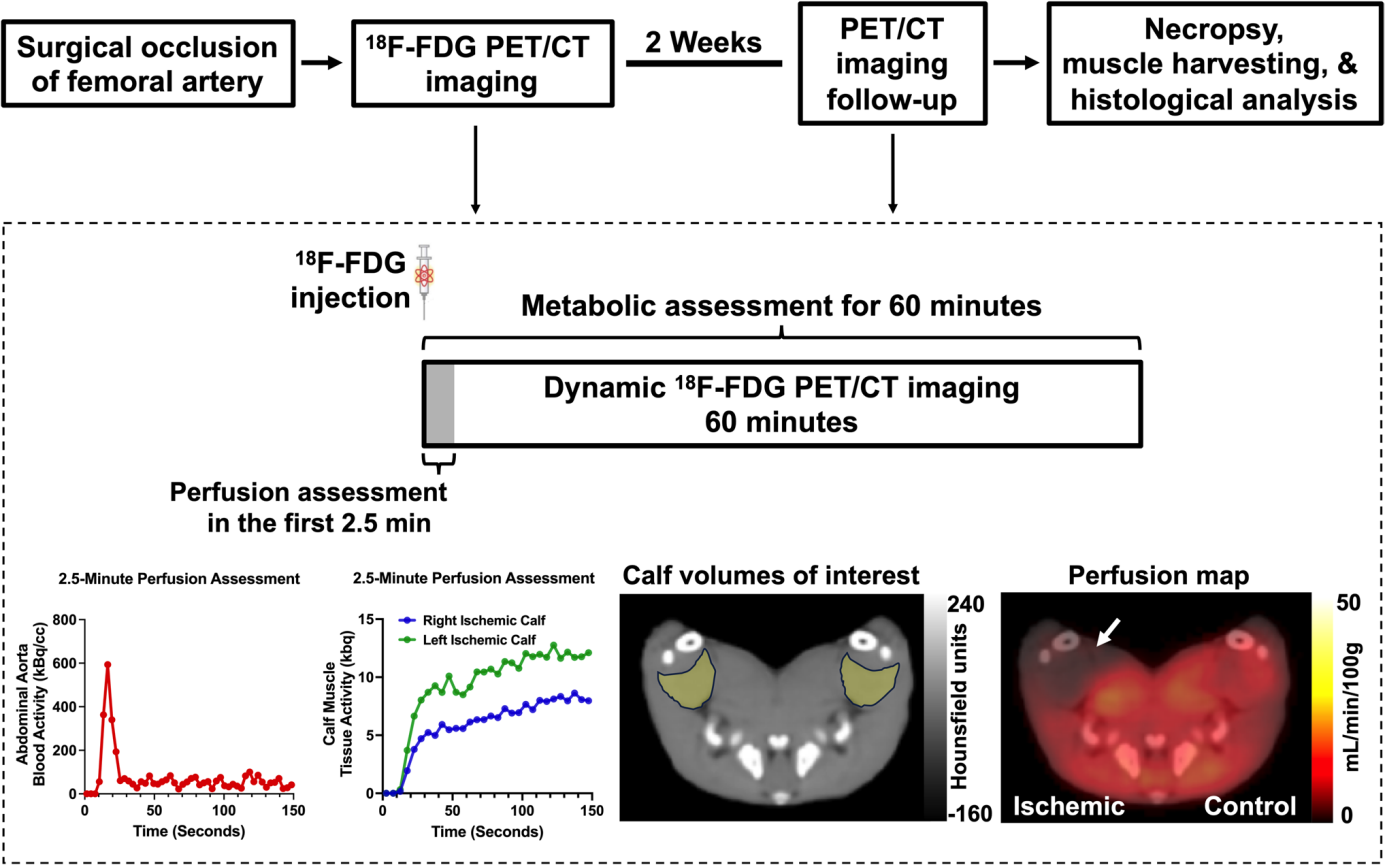
Experimental workflow for performing dual monitoring of lower extremity skeletal muscle perfusion and metabolism using ^18^F-FDG PET/CT imaging. Representative schematic displaying the study timeline, representative time activity curves for arterial blood and calf muscle tissue for the first 2.5 min of PET data acquisition, calf muscle segmentation, and resulting skeletal muscle perfusion map after 1-tissue compartment modeling. The perfusion deficit induced by unilateral femoral artery occlusion and localized to the calf is shown on fused PET/CT imaging

Dynamic ^18^F-FDG PET/CT imaging was performed on the day of femoral artery occlusion and 2 weeks after occlusion using a hybrid PET/CT system (Discovery 690, GE Healthcare, Chicago, IL). Seven of eight animals underwent serial dynamic PET imaging in list-mode for 60 min, and one of the eight animals underwent serial dynamic PET imaging for 2.5 min. CT images were acquired with voxel dimensions of 1.37 × 1.37 mm and slice thickness of 3.27 mm, at 120 kVp and 300 mA, for attenuation correction and calf muscle segmentation. The PET/CT imaging study design is outlined in Fig. 1.

The 60-min dynamic PET scan was started in list mode immediately before intravenous injection of ^18^F-FDG (196.8 ± 7.5 MBq), with ^18^F-FDG dose and saline flush administered over a 20-s duration. The animal’s lower abdomen and hindlimbs were positioned in the PET camera’s field-of-view (FoV) (~ 15.7 cm axial FoV) to ensure coverage of both the abdominal aorta and calf muscles. 1 ml of arterial blood was collected at baseline (prior to imaging) and then continuously sampled every 5 s for the first 2.5 min of PET imaging at a constant rate (12 ml/min) using a Harvard peristaltic pump (Harvard Apparatus, South Natick, MA). Approximately 0.5 ml of each sample was used to measure whole blood radioactivity while the other 0.5 ml of blood was centrifuged at 2000 g for 15 min to acquire plasma radioactivity. Whole blood and plasma samples were weighed, counted, and corrected for radioactive decay using a gamma well counter (WIZARD^2^, PerkinElmer, Waltham, MA). Whole blood and plasma time activity curves (TACs) were then obtained, as well as plasma-to-whole blood ratios for each arterial sample. In addition to arterial blood sampling for gamma counting, plasma samples were analyzed for glucose concentration at baseline (prior to initiation of PET imaging) and every 10 min during image acquisition (i.e., 10-, 20-, 30-, 40-, 50-, and 60-min time point) to evaluate stability of plasma glucose during the 60-min PET acquisition. All measurements of plasma glucose concentration were assessed using a commercially available blood glucose monitor (AlphaTRAK 2; Zoetis Inc., NJ, USA).

### PET/CT Image Reconstruction and Processing

All dynamic PET data was reconstructed using 2 iterations and 32 subsets of the ordered subset expectation and maximization (OSEM) algorithm with trans-axial full width at half maximum (FWHM) Gaussian filter equal to 4.5 mm. PET image volumes were preprocessed using Hounsfield-unit based attenuation correction. The PET image reconstruction matrix was 192 × 192 pixels, with voxel dimensions of 3.65 × 3.65 mm and a slice thickness of 3.27 mm. To address potential limitations of partial volume and spill-over effects for the image-derived arterial input function (AIF), arterial blood sampled during dynamic PET imaging was used to generate correction factors for the image-derived plasma AIF using recently published methods in the same porcine model of hindlimb ischemia [10]. All aspects of PET/CT image processing were performed using commercially available software (PMOD Technologies LLC, Fallanden, Switzerland).

For evaluation of skeletal muscle perfusion, the first 2.5 min of the PET list data was reconstructed in 3-s frames to obtain the AIF from the abdominal aorta, and 5-s frames for analysis of ^18^F-FDG kinetics in the bilateral calf muscles (Fig. 1). Calf perfusion values were calculated using a 1-tissue compartment kinetic model that incorporated the corrected image-derived plasma AIF, skeletal muscle TACs, and blood volume as a fitting parameter. First-pass extraction of ^18^F-FDG was assumed to be 100% in peripheral skeletal muscle with zero venous outflow. While it is understood that first-pass extraction of ^18^F-FDG is not 100% due to ^18^F-FDG not being freely diffusible, this assumption for a 1-tissue compartment model was previously used when validating dynamic ^18^F-NaF PET imaging as an approach for quantifying skeletal muscle perfusion [10] and by Mullani et al. [14] when validating dynamic ^18^F-FDG PET as a method for quantifying first-pass tumor perfusion. To adjust for variability in hemodynamic loading conditions, perfusion values were normalized by rate pressure product (RPP) by multiplying the perfusion value by the reference RPP for the study cohort and then dividing by the individual RPP at the time of PET perfusion imaging. Details of this PET image processing workflow were recently described and validated in the same porcine model of hindlimb ischemia using ^18^F-NaF [10].

For evaluation of skeletal muscle metabolism, the entire 60-min PET list data was reconstructed using the following sequence: 6 frames of 20 s each, followed by 3 frames of 60 s each, 3 frames of 300 s each, and 4 frames of 600 s each, as previously described by Pande et al. [11]. The overall rate of ^18^F-FDG uptake (Ki) of the ischemic and control calf was quantified from PET imaging with a 3-tissue compartment model developed by Bertoldo et al. [15] using an image-derived AIF and calf muscle TACs. The metabolic rate of glucose (MRGlu) in the calves was then calculated by multiplying the image-derived Ki by the plasma glucose concentration and correcting for the relative metabolism of ^18^F-FDG versus metabolism of true glucose using the lumped constant, which was assumed to be 1.2 for skeletal muscle based on the findings of Kelley et al. [16]. The MRGlu for was ultimately expressed as $\mu$ mol/min/kg of tissue.

### Quantification of Peripheral Microvessel Remodeling

Following completion of PET/CT imaging at 2 weeks post-occlusion, animals were euthanized via intravenous administration of euthasol (1 ml/4.5 kg), and muscle samples (~ 20 g) were harvested from the gastrocnemius muscle of the bilateral calves in 7 of 8 pigs to assess the relationship between calf muscle angiogenesis and recovery of calf muscle perfusion 2 weeks after arterial occlusion. Muscle tissue sampling sites were standardized for the ischemic and control hindlimbs. Samples were fixed with 4% paraformaldehyde, paraffin embedded and sectioned at 4-μm thickness. Sections were stained with primary Anti-CD31 antibody (ab28364, Abcam, Cambridge, U.K.) and secondary Alexa Fluor 580 IgG goat anti-rabbit antibody (A-11011, Thermo Fisher Scientific). All sections were then imaged on a fluorescent microscope (BZ- × 800, Keyence, Itasca, IL) and image quantification was performed as previously described using ImageJ (National Institutes of Health) [10]. Capillary density of the gastrocnemius calf muscle was expressed as capillary-to-muscle fiber ratios.

### Statistical Analysis

Serial changes in PET-derived measures of muscle perfusion and muscle metabolism were evaluated using a repeated measures analysis of variance (ANOVA) with a linear mixed effect model, with animal included as random effect. Model residuals were assessed for normality using the Shapiro–Wilk test and Q-Q plots. For outcomes that satisfied normality assumptions, pairwise comparisons were performed using estimated marginal means with Tukey adjustment for multiple comparisons. Paired comparisons not meeting normality assumptions (i.e., MRGlu) were performed using the Wilcoxon signed rank test with Holm-Sidak adjustment for multiple comparisons. Differences in plasma glucose between imaging sessions, as well as differences in capillary density between control and ischemic hindlimb calf muscles were evaluated using paired t-tests. A p-value of < 0.05 was considered statistically significant for all analyses. Statistical analyses were performed using R, version 4.3.0 in R Studio, and Prism for macOS, version 9.3.0 (GraphPad Software, LLC).

## Results

### ^18^F-FDG PET/CT Imaging of Skeletal Muscle Perfusion

**Fig. 2:**
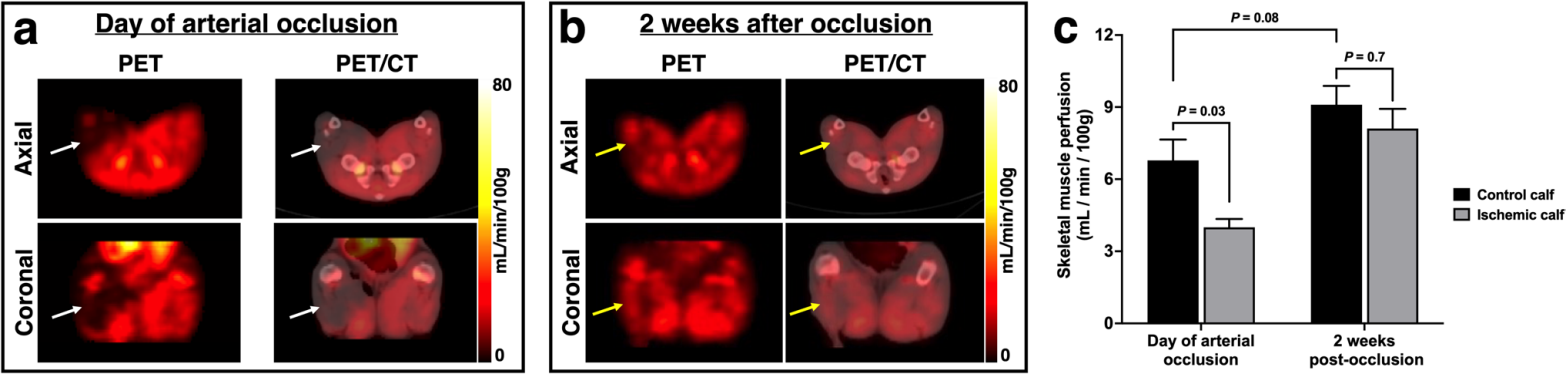
Serial quantification of calf muscle perfusion in a porcine model of hindlimb ischemia. Dynamic ^18^F-FDG PET imaging demonstrated (**a**) a qualitative reduction in calf muscle perfusion in response to unilateral femoral artery occlusion (white arrows) and (**b**) perfusion recovery 2 weeks after arterial occlusion (yellow arrows). (**c**) PET image analysis confirmed a significant quantitative decrease in absolute measures of calf muscle perfusion in the ischemic hindlimb that returned to control levels 2 weeks later. Data are shown as means ± SEM. N = 8 pigs

One-tissue compartment modeling of the first 2.5 min of dynamic PET data allowed for quantification of significant reductions in calf muscle perfusion in response to unilateral femoral artery occlusion (ischemic calf, 4.0 ± 1.0 mL/min/100 g versus control calf, 6.8 ± 2.5 mL/min/100 g; *P* = 0.03) (Fig. 2a). PET-derived measures of calf muscle perfusion returned to control levels 2 weeks after arterial occlusion (ischemic calf, 8.1 ± 2.3 mL/min/100 g versus control calf, 9.1 ± 2.2 mL/min/100 g; *P* = 0.7; Fig. 2b-c).

In the development of an optimal AIF for one-tissue compartment modeling and perfusion computation, TACs from the abdominal aorta were generated (Fig. 1). The best fitting value was an $\alpha$ value of 0.8 for the partial volume correction factor and a $\beta$ value of 1 for the spill-over correction factor. The plasma-to-whole blood ratio did not change in arterial blood samples collected in the first 2.5 min of dynamic PET imaging. Thus, the mean plasma-to-whole blood ratio (1.30 ± 0.04) was used to correct whole-blood TACs to plasma TACs for PET perfusion image analysis.

### ^18^F-FDG PET/CT Imaging of Skeletal Muscle Metabolism

**Fig. 3:**
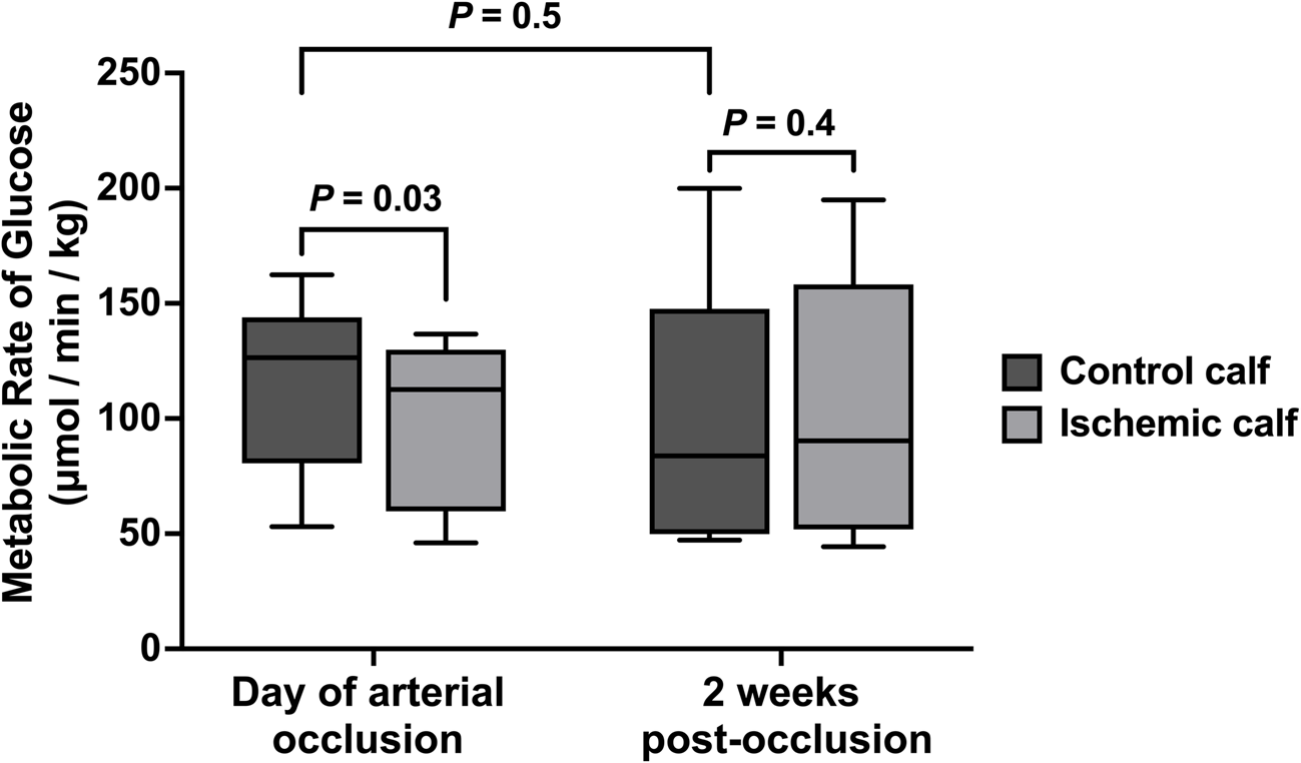
Serial evaluation of calf muscle metabolism in a porcine model of hindlimb ischemia using dynamic ^18^F-FDG PET/CT imaging. Quantitative PET/CT image analysis demonstrated that the metabolic rate of glucose was significantly reduced in the calf muscle of the ischemic limb on the day of arterial occlusion and recovered to control levels 2 weeks later. Lines in box plot represent medians, lower and upper box limits represent 25th and 75th percentiles, and whiskers extend to the minimum and maximum values. N = 7 pigs

Three-tissue compartment modeling of the 60-min PET dataset revealed that the overall rate of ^18^F-FDG uptake (Ki) was significantly reduced in the ischemic calf compared to the control calf on the day of femoral artery occlusion (0.014 ± 0.005 ml/min/g versus 0.017 ± 0.005; *P* = 0.004) and recovered to control levels 2 weeks after arterial occlusion (ischemic calf, 0.016 ± 0.008 ml/min/g versus control calf, 0.016 ± 0.007 ml/min/g; *P* = 0.5). Plasma glucose concentrations did not differ during or across the first and second PET/CT imaging sessions (first scan, 8.3 ± 1.2 mmol/L versus second scan, 8.0 ± 0.8 mmol/L; *P* = 0.5). The MRGlu in lower extremity skeletal muscle was significantly reduced in the ischemic calf compared to the control calf on the day of arterial occlusion (97.6 ± 35.3 $\mu$ mol/min/kg versus 117.2 ± 38.3 $\mu$ mol/min/kg; *P* = 0.03) (Fig. 3). Measures of MRGlu in calf skeletal muscle recovered to control levels two weeks after arterial occlusion (ischemic calf, 111.3 ± 58.6 $\mu$ mol/min/kg vs. control calf, 106.7 ± 58.5 $\mu$ mol/min/kg; *P* = 0.4) (Fig. 3).

### Skeletal Muscle Microvascular Density

**Fig. 4:**
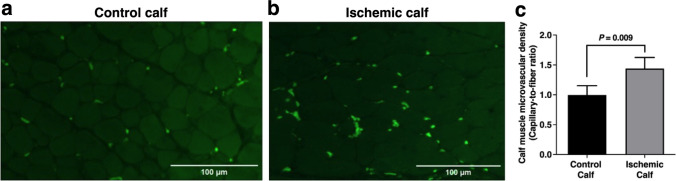
Analysis of calf muscle microvascular remodeling 2 weeks after surgical ligation of the femoral artery. Representative immunofluorescence images of CD31 in the gastrocnemius muscle sampled from the (**a**) control and (**b**) ischemic hindlimb showed a qualitative increase in capillary density in the ischemic calf compared to the control calf. (**c**) Quantitative image analysis demonstrated that capillary density significantly increased in the ischemic versus control hindlimb, indicating that recovery of calf muscle perfusion and metabolism 2 weeks after arterial occlusion was associated with compensatory microvascular remodeling. Data are shown as means ± SEM. N = 7 pigs

Qualitative assessment of immunofluorescent images stained for CD31 revealed an increase in microvascular density of the ischemic versus control hindlimb (Fig. 4). Quantitative image analysis further demonstrated that relative capillary density (expressed as capillary-to-muscle fiber ratio) was significantly increased for the gastrocnemius calf muscle 2 weeks after induction of hindlimb ischemia (ischemic calf ratio, 1.4 ± 0.5 versus control calf ratio, 1.0 ± 0.4; *P* = 0.009) (Fig. 4c).

## Discussion

The present study is the first to demonstrate the feasibility of multiparametric ^18^F-FDG PET imaging for dual quantification of skeletal muscle perfusion and metabolism, in addition to being the first to monitor serial changes in both physiological parameters in the setting of limb ischemia. Dynamic ^18^F-FDG PET data from the first 2.5 min following ^18^F-FDG injection allowed for quantification of significant reductions in resting skeletal muscle perfusion within the calf of the occluded hindlimb of pigs and quantified subsequent perfusion recovery in the calf of the same limb 2 weeks later that coincided with significant increases in calf muscle microvascular density. In addition to non-invasively monitoring serial changes in lower extremity perfusion, single-dose administration of ^18^F-FDG allowed for quantification of serial alterations in skeletal muscle metabolism during the same PET acquisition. The findings of this study are significant, as they suggest that dynamic ^18^F-FDG PET can provide an efficient workflow for quantifying vascular occlusion-related alterations in resting skeletal muscle perfusion with one of the world’s most widely used and accessible PET radionuclides. Given that ^18^F-FDG PET allowed for quantification of muscle perfusion within the first 2.5 min after dose injection, the present study also reveals that a longer duration PET scan (i.e., 1-h) can be utilized to provide measures of both skeletal muscle perfusion and metabolism, thus offering a comprehensive imaging framework for quantifying the physiological consequences of limb ischemia.

Prior nuclear medicine studies have shown that SPECT imaging can quantify relative perfusion defects in the lower extremities of patients with PAD [3, 5, 6], while studies using dynamic PET imaging have demonstrated the ability to quantify absolute measures of skeletal muscle perfusion in PAD [7–9]. However, previous dynamic PET investigations in the setting of PAD have primarily used short half-life radioisotopes, such as oxygen-15-water (2.04 min), which has likely limited the widespread translatability of PET imaging to the PAD patient population due to the need for onsite radioisotope production and rapid administration by well-trained radiopharmacy staff. Therefore, our team recently validated the first dynamic PET imaging approach that uses 1-tissue compartment modeling of a commercially available ^18^F-labeled radionuclide (^18^F-NaF, 109.7 min half-life) for quantifying skeletal muscle perfusion [10]. The present study builds on this prior PET imaging work by demonstrating that a more readily available ^18^F-labeled radionuclide (i.e., ^18^F-FDG) can also be used to provide similar physiologically relevant measures of resting skeletal muscle perfusion in the setting of limb ischemia. The perfusion values in the present study closely align with those reported in our previously published work using dynamic ^18^F-NaF PET imaging, which was first validated in an identical porcine model of hindlimb ischemia using the gold-standard microsphere approach and then translated to patients with PAD [10]. Additionally, the present study demonstrated similar improvements in microvascular density 2 weeks after femoral artery occlusion as in our prior porcine study, and these compensatory changes in microvascular remodeling aligned with the timeline for recovery of calf perfusion and metabolism.

In addition to quantifying skeletal muscle perfusion, the present study demonstrated for the first time that perfusion assessment could be performed in combination with metabolic assessment during the same dynamic ^18^F-FDG PET scan. Additionally, the present work demonstrated for the first time the use of ^18^F-FDG PET imaging for monitoring serial changes in both skeletal muscle perfusion and metabolism in the setting of limb ischemia. Although prior work has used dynamic ^18^F-FDG PET imaging to quantify skeletal muscle metabolism and demonstrate impairments in glucose uptake in the setting of PAD [11], the novel evaluation of two key physiological parameters that are influenced by skeletal muscle ischemia may allow for expanded applications of ^18^F-FDG PET for musculoskeletal disorders in the future and provide a more robust functional tool for vascular medicine clinicians to assess PAD pathophysiology. In the evaluation of skeletal muscle metabolism, we found that glucose metabolism in the occluded hindlimb was significantly impaired after arterial occlusion and subsequently recovered in a similar manner as perfusion measures 2 weeks after vascular occlusion. The MRGlu values (i.e., ~ 100 $\mu$ mol/min/kg) obtained in the present study for peripheral skeletal muscle of healthy young pigs were comparable to MRGlu values previously reported for skeletal muscle of healthy human subjects (i.e., ranging approximately 60–120 $\mu$ mol/min/kg) when blood glucose levels were maintained at similar levels as those measured in our porcine model (i.e., ~ 5 mmol/L) [17, 18].

Although this study demonstrated the feasibility of serially measuring skeletal muscle perfusion and metabolism with ^18^F-FDG PET/CT imaging, it should be noted that the animals used in the present study were healthy young pigs undergoing an active growth phase, which have well-documented compensatory vascular remodeling and perfusion recovery in response to peripheral artery occlusion [10, 12]. Therefore, future translation of this imaging approach to a pre-clinical model of chronic limb ischemia or patients with PAD is warranted to fully assess the utility of this approach in the setting of chronic disease. Additionally, no microsphere validation studies were performed in combination with the chronic PET perfusion imaging studies; however, the perfusion values obtained in the present study closely aligned with those in prior work that used an identical porcine model, an ^18^F-labeled radionuclide (^18^F-NaF), microsphere validation, and identical PET image processing pipeline.

## Conclusions

The current study demonstrates that multiparametric PET/CT imaging with a single dose injection of ^18^F-FDG is a feasible approach for quantifying physiologically relevant measures of skeletal muscle perfusion and metabolism in the setting of limb ischemia. Of note, ^18^F-FDG does not require on-site cyclotron production and is one of most readily available radionuclides for PET imaging, thereby enabling seamless translation of this approach in the future to patients with PAD. Thus, comprehensive assessment of skeletal muscle physiology with ^18^F-FDG PET/CT imaging is a practical approach that may provide functional insight into tissue viability and limb outcomes in the future for patients with underlying ischemia and other musculoskeletal disorders.

## Data Availability

Data are available from the authors upon reasonable request.
